# Overweight is not associated with cortical thickness alterations in children

**DOI:** 10.3389/fnins.2015.00024

**Published:** 2015-02-04

**Authors:** Rachel J. Sharkey, Sherif Karama, Alain Dagher

**Affiliations:** ^1^McConnell Brain Imaging Centre, Montreal Neurological Institute, McGill UniversityMontreal, QC, Canada; ^2^Douglas Mental Health University Institute, McGill UniversityMontreal, QC Canada

**Keywords:** obesity, cortical thickness, cortical development, MRI, adolescence, childhood, gray matter, body mass index

## Abstract

**Introduction:** Several studies report an association between body mass index (BMI) and cortical thickness in adults. Some studies demonstrate diffuse cortical thinning in obesity, while others report effects in areas that are associated with self-regulation, such as lateral prefrontal cortex.

**Methods:** This study used multilevel modeling of data from the NIH Pediatric MRI Data Repository, a mixed longitudinal and cross-sectional database, to examine the relationship between cortical thickness and body weight in children. Cortical thickness was computed at 81,942 vertices of 716 MRI scans from 378 children aged between 4 and 18 years. Body mass index Z score for age was computed for each participant. We performed vertex-wise statistical analysis of the relationship between cortical thickness and BMI, accounting for age and gender. In addition, cortical thickness was extracted from regions of interest in prefrontal cortex and insula.

**Results:** No significant association between cortical thickness and BMI was found, either by statistical parametric mapping or by region of interest analysis. Results remained negative when the analysis was restricted to children aged 12–18.

**Conclusions:** The correlation between BMI and cortical thickness was not found in this large pediatric sample. The association between BMI and cortical thinning develops after adolescence. This has implications for the nature of the relationship between brain anatomy and weight gain.

## Introduction

Cortical Thickness is both a marker of neurological development and a reflection of cortical function (Jernigan et al., 1991; Pfefferbaum et al., 1994; Reiss et al., 1996; Giedd et al., 1999a,b). Body weight is one factor which has been associated with alterations in cortical thickness. Obesity and overweight has been associated with reduced global gray matter volume in young adults, healthy older adults, and older adults with Alzheimer's disease (Pannacciulli et al., 2006, 2007; Taki et al., 2008; Ho et al., 2010; Hassenstab et al., 2012; Maayan et al., 2012; Yokum et al., 2012; Brooks et al., 2013; Horstmann et al., 2013, 2011; Marqués-Iturria et al., 2013). While some studies report diffuse cortical thinning in obese individuals, others have found reduced cortical thickness specifically in regions associated with self-control and reward (Pannacciulli et al., 2006; Hassenstab et al., 2012; Maayan et al., 2012; Yokum et al., 2012; Marqués-Iturria et al., 2013). These regions include the prefrontal cortex (Ho et al., 2010; Marqués-Iturria et al., 2013), specifically the dorsolateral prefrontal cortex (Brooks et al., 2013), the orbitofrontal cortex (OFC) (Maayan et al., 2012), and the dorsal anterior cingulate cortex (Hassenstab et al., 2012).

The direction of the causal relationship between cortical thickness and body weight is not, however, entirely clear. Thinner gray matter in areas related to self-regulation and motivation could result in excess food intake, but it is also possible that metabolic factors related to excess weight could lead to reduced cortical thickness. A study by Taki et al. found that body weight was negatively correlated with brain volume in men, but not women. Since women tend to have more subcutaneous fat while men tend to store visceral fat, this would be in line with the hypothesis that increased inflammatory proteins associated with visceral fat play a role in decreased cortical thickness in obesity and overweight (Taki et al., 2008). Alternatively, dysregulation of insulin and leptin, both of which act as neurotrophic factors, has also been associated with reduced frontal cortical thickness in both humans and animal models (Pannacciulli et al., 2006). Humans and animals with leptin mutations have also been found to have reduced cortical thickness, which can be reversed with exogenous leptin treatment (Pannacciulli et al., 2007).

The relationship between obesity and brain volume in older adolescents is less well established. In one study, obese adolescents (ages 14–21) were found to have reduced orbitofrontal cortex volume, high scores on all domains of the Three Factor Eating Questionnaire and impaired cognitive task performance, notably on tests of inhibitory control. This was hypothesized to reflect a relationship between body weight, OFC function and a tendency to disinhibited eating (Maayan et al., 2012). A second study in women with an average age of 18 years, however, found a global decrease in gray matter in obese individuals, as compared to those classified as lean or overweight (Yokum et al., 2012). The same study, however, found no significant local differences in gray matter volume in a series of regions of interest including the insula, post-central gyrus, caudate, putamen and frontal gyri, between obese, overweight and lean individuals (Yokum et al., 2012).

Cortical thickness in children has been found, overall, to decrease with age, peaking around 4, and decreasing until as late as 30 years of age (Jernigan et al., 1991; Pfefferbaum et al., 1994; Reiss et al., 1996). Different brain regions mature at different rates, but, overall the brain matures from back to front, with the prefrontal cortex being one of the last areas to develop.

Finally, there is limited information on the relationship between BMI and brain volume or cortical thickness in children. The main goal of this study is to assess the relationship between body weight and cortical thickness in children using data from the NIH Pediatric MRI Data Repository, a mixed cross-sectional and longitudinal database of brain development in healthy, normally developing children (Evans, 2006).

## Methods

### Sampling and dataset selection

Data were taken from the NIH Pediatric MRI Data Repository (Evans, 2006). The Repository contains data from the NIH MRI Study of Normal Brain Development. Objective One of this study consisted of 431 children between the ages of 4 and 18 years. Recruitment and scanning occurred at six sites in the United States (US). The institutional review boards at each institution approved the study protocol and both parental consent and participant assent were obtained before testing. Participants were recruited by geocoded mailed survey to reflect the demographic distribution of the US population to prevent any bias and ensure that the sample was representative of the distribution of age, sex, race and socioeconomic status of the zip codes where recruitment occurred (according to 2000 US Census Data). Participants were screened to rule out neurological illness or trauma, axis I psychiatric illness or a family history of the same, language disorders or substance abuse disorders as well as prematurity, exposure to toxins *in utero*, or most birth complications. Participants with IQ scores below 70 or Child Behavior Checklist (CBCL) subscale score of over 70 were also excluded. The complete recruitment protocol for the NIH MRI Study of Normal Brain Development can be found at http://pediatricmri.nih.gov/nihpd/info/. Each participant was scanned three times, 2 years apart. Following collection, imaging and behavioral data was stored on a customized database at the Montreal Neurological Institute (MNI).

For this study, only timepoints where age, gender, height and weight (used to calculate BMI) were available and where cortical thickness data passed quality control, were used. This resulted in 378 subjects and 716 datapoints (395 female, 109 overweight, 95 obese according to the CDC guidelines for children and adolescents). Racial distribution in the original sample was 11% Black or African American, 12% Hispanic, 72% White, and 5% Other (Evans, 2006). At time point one racial distribution in the subset used in this study was 9.9% Black or African American, 12.5% Hispanic, 76.0% White, and 14% Other. At the second time point racial distribution was 10.5% Black or African American, 10.5% Hispanic, 75.7% White, and 13.8% Other. At the third time point the distribution was 10.1% Black or African American, 13% Hispanic, 74.5% White, and 15.4% Other.

### MRI acquisition and image processing protocol

The full MRI Acquisition protocol for the NIH MRI Study of Normal Brain Development can be found at http://pediatricmri.nih.gov/nihpd/info/. Briefly, subjects underwent a sagittal T1 weighted 3D RF-spoiled gradient echo sequence covering the entire head with slice thickness of between 1 and 1.5 mm. Shorter alternate sequences with 3 mm slice thickness were used for subjects who had difficulty holding still for the scan but none of these were used for the current analyses as the lower spatial resolution is deemed inadequate for precise cortical thickness estimation.

The T1 weighted image was then processed using the CIVET (version 1.1.12) image processing pipeline (Ad-Dab'bagh et al., 2006) to compute gray and white matter boundaries and surfaces, which were then used to calculate cortical thickness. Images were first linearly registered to MNI space based on the ICBM152 template. N3 was used to correct for non-uniformity and INSECT, a neural net classifier, was used to classify all voxels into gray matter, white matter and cerebrospinal fluid. CLASP was used to generate 2D inner and outer cortical surfaces, which are formed from deformable polygon meshes with 81942 vertices (where the cortical thickness is calculated). The meshes were then registered to the ICBM152 template to ensure the vertices line up between participants. Data were smoothed using a surface based 20 mm Gaussian kernel. The full details of the CIVET pipeline can be found at http://www.bic.mni.mcgill.ca/ServicesSoftware/CIVET.

### BMI calculations

Healthy BMI in children varies substantially with age, meaning that BMI cannot be compared directly between subjects in our database. Instead a percentile for age was calculated based on a standardized growth curve. BMI, BMI Percentile for age and BMI Z-Score for age were calculated by inputting height, weight and age data into EpiInfo 7 from the Centers for Disease Control (CDC) and Prevention (http://wwwn.cdc.gov/epiinfo/7/). The Z-Scores and percentiles were calculated based on the official CDC growth curves.

### Cortical thickness analysis

Statistical analysis of the cortical thickness data was conducted using SurfStat (Worsley et al., 2004), a statistical toolbox running in Matlab R2012b (The Mathworks, Inc.). A mixed-effects model of the effects on cortical thickness of age, gender, BMI Z-Score for age and scanner, as fixed effects, and subject identity as a random effect, was created using the following model:

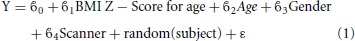



Where Y is cortical thickness, 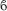
_0_ represents the y-intercept, 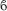
_1–4_ are the regression coefficients of the variables and ε is an error term. The regression was run at each of the 81924 vertices. A 0.05 false discovery rate (FDR) was used to account for multiple comparisons. This model was run on the full dataset and also on a subset of all subjects older than age twelve.

Two interaction models were also run. In the first, an interaction term between age and BMI Z Score was added to the original model to account for differing effects of BMI on cortical thickness at different ages:

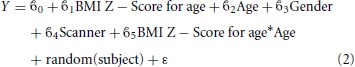



In the second interaction model, the three way interaction between BMI Z Score, age and gender was tested using the following model:

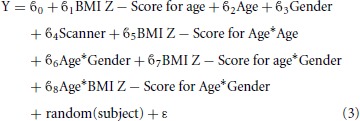



This model sought to account for both potential differences in the effect of BMI on cortical thickness with age, and how the effect might change with gender.

Mean cortical thickness was also calculated for each timepoint and a correlation between mean cortical thickness and BMI Z Score for Age was run.

### Region of interest analysis

Average cortical thickness values for each participant at each time point were extracted for five regions of interest (ROI) thought to be involved in appetite control. The areas, defined by the AAL atlas (Tzourio-Mazoyer et al., 2002), were the left and right insula, the left and right superior frontal dorsolateral region, the left and right superior orbital frontal region, the left and right middle frontal orbital region and the left and right inferior orbital frontal region. These regions were chosen because their gray matter volume has been previously identified as predicting both BMI (Horstmann et al., 2013, 2011) and personality measures that are predictive of BMI (Deyoung et al., 2010; Vainik et al., 2013) in young adults.

The simple, interaction-free multilevel model used earlier (Equation 1) was evaluated using SPSS Version 20 (IBM Corp. Armonk, NY) for each ROI independently.

### Analysis of behavioral effects

Separate multilevel models of cognitive and demographic effects on BMI Z-Score for age were conducted in SPSS 20. All models accounted for the effects of age and gender. Models were created for the Wechsler Abbreviated Scale of Intelligence IQ score, CBCL attention subscale score, Cambridge Neuropsychological Test Automated Battery Intra-Extra Dimensional (IED) Set Shift score and household income. Household income data in the NIH Pediatric Data Repository was binned into brackets, each of which was assigned an ordinal number. For this analysis the household income data was treated as pseudocontinuous. IED was included as a measure of executive function, different aspects of which have been shown to predict obesity in adults (Vainik et al., 2013).

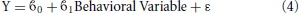



Here Y represents BMI Z-Score for Age, 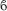
_0_ represents the y-intercept, 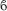
_1_ is the regression coefficient of the variable and ε is the error term. WASI and IED were found to be redundant in the individual models and were removed, so they were not included in the complete model. Based on the results of the single variable analysis a complete model of all the non-redundant variables was created.

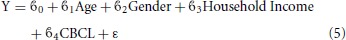



## Results

**Figure 1:**
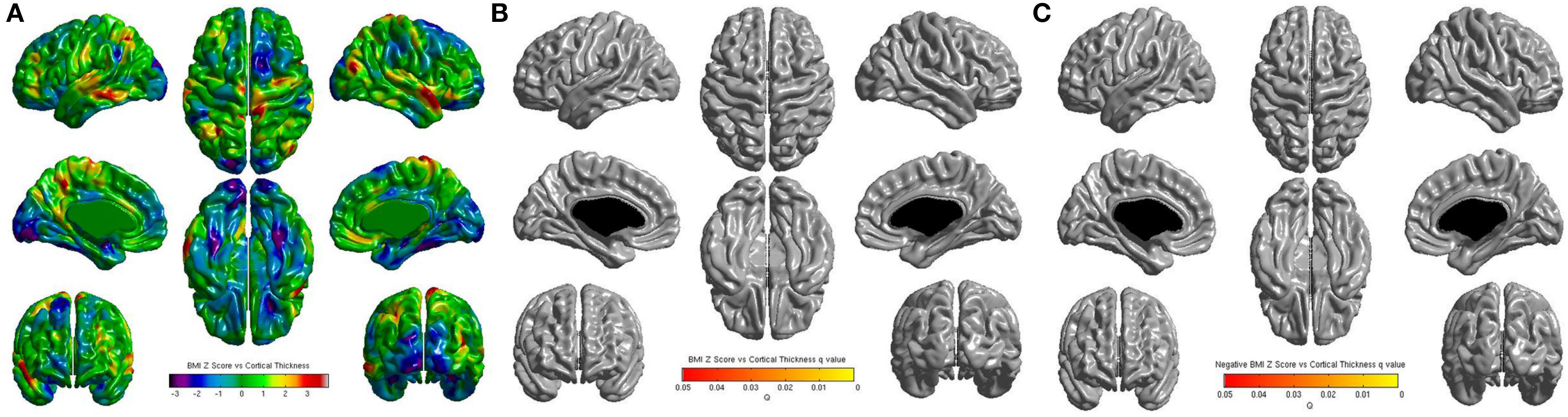
**(A)** Unthresholded correlations between BMI Z-Score for age and cortical thickness across the entire brain, shown here for comparison. Typically *T*-values of around 4 or greater are needed to reach significance. **(B)** Positive correlations between BMI Z-Score for age and cortical thickness across the entire brain corrected for multiple comparisons with FDR *q* = 0.05 **(C)** Negative correlations between BMI Z-Score for age and cortical corrected for multiple comparisons with FDR *q* = 0.05.

**Figure 2:**
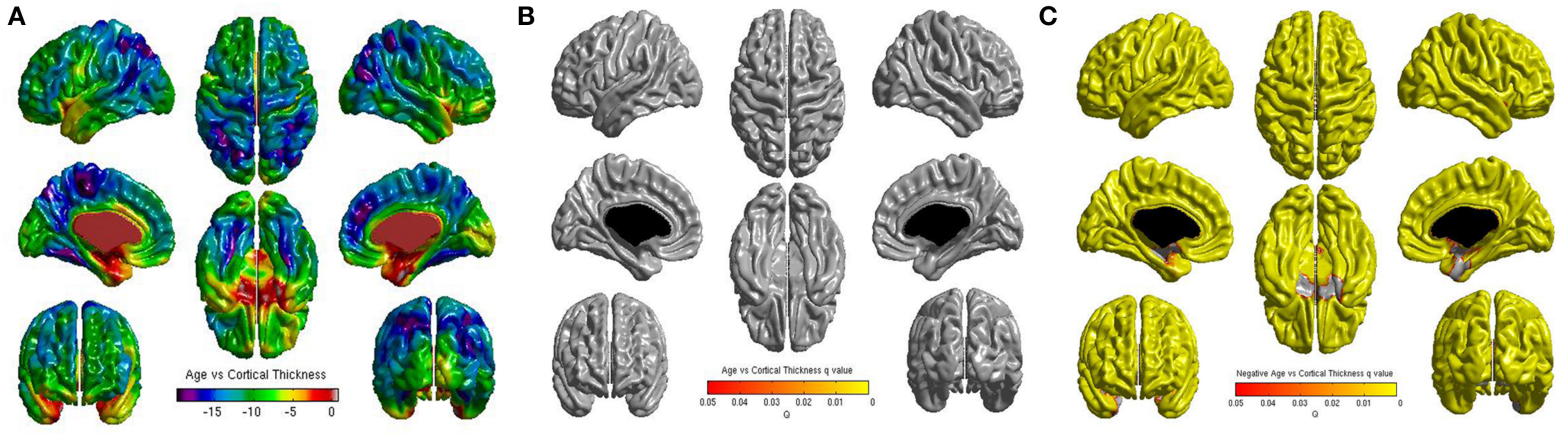
**(A)** Unthresholded correlations between age and cortical thickness across the entire brain, shown here for comparison. **(B)** Positive correlations between age and cortical thickness across the entire brain corrected for multiple comparisons with FDR *q* = 0.05. **(C)** Negative correlations between age and cortical thickness across the entire brain corrected for multiple comparisons with FDR *q* = 0.05.

**Figure 3:**
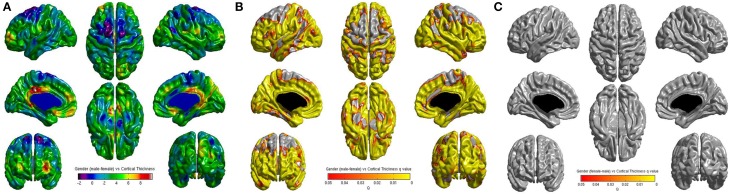
**(A)** Unthresholded correlations between gender (male—female) and cortical thickness across the entire brain, shown here for comparison. **(B)** Positive correlations (male—female) between gender and cortical thickness across the entire brain corrected for multiple comparisons with FDR *q* = 0.05. **(C)** Negative correlations (female—male) between gender and cortical thickness across the entire brain corrected for multiple comparisons with FDR *q* = 0.05.

**Figure 4:**
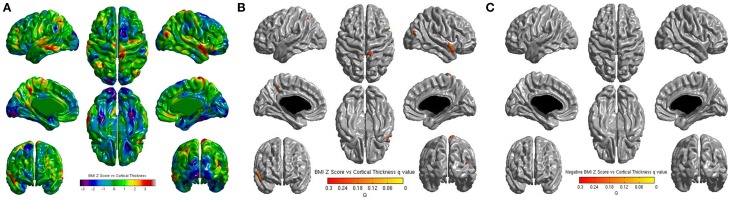
**(A)** Unthresholded correlations between BMI Z-Score for age and cortical thickness across the entire brain, shown here for comparison. Typically *T*-values of around 4 or greater are needed to reach significance. **(B)** Positive correlations between BMI Z-Score for age and cortical thickness across the entire brain corrected for multiple comparisons with FDR *q* = 0.3 **(C)** Negative correlations between BMI Z-Score for age and cortical corrected for multiple comparisons with FDR *q* = 0.3.

No significant correlations were found between cortical thickness and BMI Z Score using FDR multiple comparison correction (Figure 1). A strong negative correlation between age and cortical thickness was found across the entire brain (Figure 2). Males had significantly thicker cortex than females across large portions of the temporal and parietal lobes and the anterior cingulate cortex (Figure 3). No significant interactions were found in either of the interaction models (Equations 2 and 3). When the FDR threshold was lowered to *q* = 0.3 for exploratory purposes there were four regions of correlation between BMI and cortical thickness in the temporal pole, precuneus, occipital lobe and primary sensory cortex. No negative correlations were found (Figure 4). No correlation between mean cortical thickness and BMI Z Score was found.

When only children over 12 (*n* = 183, 291 time points) were included in the analysis there were still no significant effects of BMI found on cortical thickness.

No significant results were found in the ROI analysis. The lowest *p*-value was *p* = 0.60, and was found in the left insula.

In the individual behavioral and cognitive models, IED and WASI were redundant, and no variables showed a significant correlation with BMI Z-score. The lowest *p*-value was *p* = 0.061, found for the effect of household income. When the remaining variables were entered into a model together, a significant negative effect of household income was found (*F* = −0.071, df = 417.785, *p* = 0.038).

## Discussion

Although there is substantial support for cortical thinning for obese adults and some for older teenagers, we found no association between BMI and cortical thickness in children in this study. A comprehensive power analysis of cortical thickness studies conducted by Pardoe et al. has found that a sample size of *n* = 50 is sufficient to detect small cortical thickness differences (less than 0.25 mm) (Pardoe et al., 2013). Since our sample is much larger than this, our dataset is likely more at risk of yielding spurious effects than failing to detect real differences. We protected against false positive results by using a false discovery rate of 0.05. The ROI analyses in areas implicated in appetite control failed to detect an effect of BMI on cortical thickness, even when uncorrected for multiple comparisons. The age and gender correlations with cortical thickness identified in this study are also in line with prior research, which supports the overall reliability of our sample and findings (Jernigan et al., 1991; Pfefferbaum et al., 1994; Giedd et al., 1999b, 2006; Nguyen et al., 2013).

The relationship between cortical thickness in prefrontal areas and BMI or weight gain is thought to result from a combination of increased incentive drive and reduced self-control leading to maladaptive decision making with respect to food choices (Horstmann et al., 2011; Vainik et al., 2013). It is most likely the case that, in younger children, other factors are responsible for determining body weight. It could be that both homeostatic satiety mechanisms and parental food choices and meal planning have more influence on food intake. For example, parental control of diet is known to be positively correlated with body weight, and it is hypothesized that rigid external control of meal timing, amount and content by parents may prevent children from learning to attend to internal hunger cues and developing appetite regulation skills. This idea has received some support from existing studies (Birch and Davison, 2001; Crossman et al., 2006; Gray et al., 2007). Differences in satiety between lean and overweight children may also be genetic. Polymorphisms in the FTO gene, which are linked to obesity, have been found to be linked to reduced sensitivity to satiety, which leads to overeating (Wardle et al., 2008). Parental food choices as an environmental factor can also be a major influence on children's weight. Parental choice determines which foods young children have access to, which can cause weight gain when children have reduced access to low energy density foods. Early food access also plays a role in which food children prefer as they develop the ability to make their own food choices (Savage et al., 2008; Scaglioni et al., 2011). Other environmental factors which have been associated with overweight and obesity in children include high parental weight, low parental education and income and, in girls, low self-esteem (Birch and Davison, 2001; Crossman et al., 2006). One major determinant of weight status for both children and adults is socioeconomic status (SES) (McLaren, 2007). In developed nations, lower SES is associated with higher rates of overweight and obesity due, presumably, to a combination of reduced access to healthy food and fewer opportunities for exercise (McLaren, 2007). Our study replicated this finding.

Impulsivity, and behaviors related to impulsivity are known to be predictive of obesity. Obese adults, especially women, have been shown to exhibit more delay discounting than their lean peers, and increased delay discounting in childhood was correlated with increased BMI as an adult in the Stanford nursery cohort (Appelhans et al., 2012; Schlam et al., 2012; Daniel et al., 2013). A major meta-analysis of neurobehavioral correlates of BMI found that body weight is most strongly influenced by the combined activity of the lateral prefrontal systems mediating executive function and self-control and the striatal network which reacts to novel and rewarding stimuli (Vainik et al., 2013). Obese women, but not obese men, have been shown to exhibit a positive correlation between gray matter volume in the putamen and right dorsolateral prefrontal cortex (Horstmann et al., 2011). A study of genetic correlates of body weight found that, in women, a polymorphism near the melanocortin-4-receptor associated with increased body weight was also associated with increased gray matter volume in the amygdala, hippocampus, orbitofrontal cortex and prefrontal cortex, all areas associated with the control of eating and food choice, and with increased scores on the disinhibition scale of the three factor eating questionnaire, and its emotional eating subscale. The same association was not found in men (Horstmann et al., 2013). These endophenotypes may be present in children but may not exert an influence on body weight until late adolescence or early adulthood, when children begin making major food choices for themselves.

If children's weight is determined primarily by external factors then the established cortical thickness effect, which is known to occur in adults, should appear over time as subjects age and develop. This could indicate that as the brain develops, variation in cortical thickness results in variation in regulation of eating behavior, that different patterns of food consumption affect cortical thickness development, or that both are altered by one or more other factors. A study following children through the transitional period from adolescence to early adulthood, rather than one which focuses on either period, would be better suited to identifying how associations between weight and cortical thickness emerge during development.

While the age range and diversity of the NIHPD sample allows for very robust conclusions about the population as a whole, it is also possible that the sample's diversity is obscuring associations that are specific to certain demographics. Follow-up studies in selective age groups may reveal specific relationships that are not seen in the general population. Another limitation of this study is that BMI-for-age was used as a measure of obesity. However, BMI does not capture individual variation in fat distribution nor does it differentiate between fat and non-fat mass. In children and adolescents BMI-for-age has been found to have a non-linear relationship with fat mass. BMI was specifically found to be less strongly correlated with fat mass in children with lower body weights (Freedman et al., 2005). Future studies using detailed analyses of body composition such as MR imaging of viscera, which were not available in this dataset, could be used to address the relationship between cortical thickness and fat mass and fat distribution more specifically (Shen et al., 2005). Determining exactly how and when the cortical thickness-obesity relationship emerges could also confirm or disprove our results more robustly, since there is still a chance, despite our high level of statistical power, that an undetectably small effect is present.

### Conflict of interest statement

The authors declare that the research was conducted in the absence of any commercial or financial relationships that could be construed as a potential conflict of interest.
